# Supplementary material for: Primary cilia in osteoblasts and osteocytes are required for skeletal development and mechano-adaptation
Source: PLoS One. 2026 Apr 2;21(4):e0346015. doi: 10.1371/journal.pone.0346015 (PMC13046246; doi:10.1371/journal.pone.0346015)
Supplement: S1 Fig — (PDF) [file pone.0346015.s001.pdf]

S1 Fig. Cortical bone μCT of IFT88 cKO mice

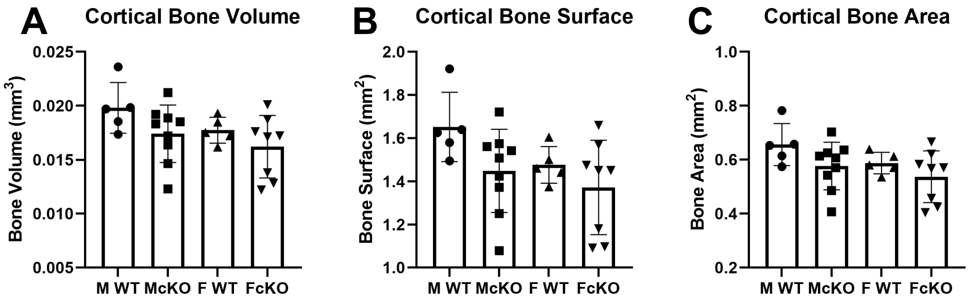

Supplementary Figure 1. Cortical analysis of OsxCre^{+}; IFT88$^{LoxP/LoxP}$ (cKO) Mice. Results collected from left femurs of male and female IFT88cKO and WT controls.
