# Supplementary material for: Primary cilia in osteoblasts and osteocytes are required for skeletal development and mechano-adaptation
Source: PLoS One. 2026 Apr 2;21(4):e0346015. doi: 10.1371/journal.pone.0346015 (PMC13046246; doi:10.1371/journal.pone.0346015)
Supplement: S2 Fig — (PDF) [file pone.0346015.s002.pdf]

S2 Fig. Cortical bone μCT of MKS5 cKO mice

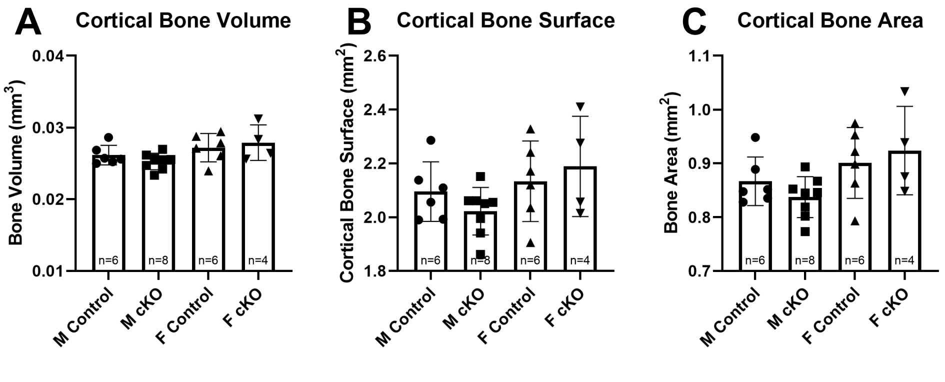

Supplementary Figure 2. Cortical analysis of Dmp1Cre^{+}, MKS5$^{LoxP/LoxP}$ (cKO) Mice. Results collected from left femurs of male and female MKS5cKO and WT controls.
