# Supplementary material for: Primary cilia in osteoblasts and osteocytes are required for skeletal development and mechano-adaptation
Source: PLoS One. 2026 Apr 2;21(4):e0346015. doi: 10.1371/journal.pone.0346015 (PMC13046246; doi:10.1371/journal.pone.0346015)
Supplement: Attachment — Submitted filename: Response to reviewers Primary cilia 2025 .docx [file pone.0346015.s004.docx]

We would like to thank the editors and reviewers for their time and effort that have put into improving our manuscript. We have addressed all the comments as detailed on our point-by-point response below. For clarity, we first list the reviewers’ comments, our responses are underneath each comment highlighted in yellow background.

Reviewer #1:

One issue concerns the genotype of the control mice. The manuscript refers to the control group simply as “WT,” but it is unclear whether these animals were true wild-type mice or Cre-negative; flox/flox littermates. Because Cre drivers such as Osx-Cre and Dmp1-Cre can independently influence skeletal phenotype, and floxed alleles may not be strictly equivalent to wild-type alleles, it is generally important that conditional knockout studies use appropriate littermate controls matched for both Cre status and floxed alleles. If such controls were used—which is likely—a brief clarification in the Methods section would greatly improve transparency.

We used Cre-positive and flox/flox negative mice as the littermate controls. We deleted “WT” but used littermate control or the controls in the text and figures.

Additionally, because the study uses two different ciliary genes (IFT88 and MKS5) in two different cell types, the rationale behind these choices could be explained more explicitly.

We add a short paragraph in the Introduction to justify two different ciliary genes knockout mice in this study on Page 6.

A concise description of how IFT88 functions in ciliogenesis and how MKS5 regulates ciliary gating at the transition zone would help readers understand how these complementary models interrogate distinct aspects of ciliary biology across the osteoblast–osteocyte lineage. Addressing these points would enhance accessibility for readers who are less familiar with ciliary molecular mechanisms.

a brief description of IFT88 and MKS5 functions was added in the Introduction on Page 6.

Major Points
1. The study applies IFT88 deletion (abolishing ciliogenesis) to osteoblasts and MKS5 deletion (impairing ciliary function) to osteocytes, but the rationale behind using different levels of ciliary disruption in different cell types is not explicitly stated. Providing a brief explanation—biological, technical, or both—would greatly improve conceptual clarity. Readers would benefit from knowing why complete loss of cilia was examined in osteoblasts, while functional impairment was examined in osteocytes.

IFT88 deletion in osteocytes has been done by a graduate student in Dr. Jacobs Lab in 2015. We have IFT88 and MKS5 floxed mice available. So, we did ITF88 in osteoblasts and MKS5 in osteocytes. We add a description in Introduction on Page 6.

2. To deepen the conceptual understanding of the study, I would like to ask the authors to consider the following two points:
What phenotype would occur if MKS5 were deleted in osteoblasts?
(Cilia would remain structurally intact but functionally impaired, potentially resulting in a milder defect in bone formation compared with IFT88 deletion.)

We agree with you if mice with MKS5 deletion in osteoblasts would result in a milder defect in bone formation compared with IFT88. However, for unknown reason, breeding between osterix-cre and MKS5 floxed mice did not go well. Because we want to focus on mechanotransduction, we focused on osteocytes.

What phenotype would occur if IFT88 were deleted in osteocytes?
(Complete loss of ciliogenesis would likely lead to a more pronounced reduction in load-induced bone formation compared with MKS5 deletion.) Adding this conceptual discussion will help clarify whether the observed phenotypes reflect gene-specific properties, cell-type specificity, or differentiation-stage dependence, thereby making the overall logic of the study more robust.

IFT88 deletion in osteocytes significantly impairs load-indued bone formation by 47% based on the graduate thesis by Dr. An M. Nguyen from Christopher Jacobs published in 2015. However, it is difficult to compare which mouse strain would lead to a more pronounced reduction in loading induced bone formation if the load-induced bone formation is not completely suppressed.

3. The manuscript would benefit from a concise discussion of possible signaling pathways affected by ciliary dysfunction—such as Wnt/β-catenin, Hedgehog, TGF-β, or mechanosensitive channels (Piezo1, TRPV4)—to strengthen the mechanistic interpretation.

Thank you for these suggestions. A concise discussion was added on Page 21. Actually, we have started working on some of those signaling molecules using live cell imaging.

4. As noted above, please specify whether the control animals were Cre-negative; flox/flox littermates rather than true wild-type mice. This clarification is important given the known effects of Cre drivers on bone biology.

Please see our response above to your general comment.

Minor Points
1. Clearly specify the statistical tests used and report exact p-values when possible.

All the exact p values were reported in the text.

2. Ensure consistent labeling and significance notation across the figures.

Thanks for the reminder. We double checked the labeling and significance notation.

Reviewer #2: 
There are two main criticisms to address here:
1) This reviewer cannot understand why the authors knocked out two different molecules (IFT88 and MKS5) to disrupt functional cilia. Are the authors sure that deletion of either gene results in the same impact on cilia (i.e., total loss of cilia and/or its function)? If so, the authors should provide experimental evidence or published facts supporting the idea. If not, it is hard to compare the two different animals.

IFT88 deletion suppresses ciliogenesis. Then there is not primary cilia on the cells. IFT88 deletion in osteocytes has been done by a graduate student in Dr. Jacobs Lab in 2015. So, we did ITF88 in osteoblasts. Without MSK5, abnormal primary cilia can form on the cells. We add a description in Introduction to justify the use of two genes knockout mouse model to examine the function of primary cilia in skeletal development and mechanotransduction on page 6.

2) The affected cell lineages in the cKO animals are unclear. For example, in the IFT88cKO mice using the Osx-Cre, chondrocytes as well as osteocytes can also be affected. So, osteoblasts may not be responsible for all the observed phenotypes including growth defect. The authors should analyze all the cell lineage for possible changes in cilia in the animals used.

The reviewer is right. Osx-cre may affect hypertrophic chondrocytes. But in this study, we used osx-cre as the littermate controls. Both cKO mice and the control mice contain osx-cre gene. Therefore, the phenotypic differences between cKO mice the control mice were caused by either IFT88 cKO or MKS5 cKO.

3) The authors should reorganize the whole logic after the two points above are clarified. Otherwise, it would be impossible to interpret the results properly.

Additional justifications were added in the Introduction on Page 6. In conclusion, functional primary cilia are necessary for skeletal development and load-induced bone formation.

MINOR POINTS
1) There are no page numbers.

Page numbers were added.

2) Table 1 was not found.

Corrected.

3) The picture in Figure 1 B is not well focused and of poor quality, which should be replaced with a better one.

A set of new images were used to replace the old ones.
