# Supplementary material for: Primary cilia in osteoblasts and osteocytes are required for skeletal development and mechano-adaptation
Source: PLoS One. 2026 Apr 2;21(4):e0346015. doi: 10.1371/journal.pone.0346015 (PMC13046246; doi:10.1371/journal.pone.0346015)
Supplement: Attachment — Submitted filename: Response to reviewers 20260201.docx [file pone.0346015.s005.docx]

We would like to thank the editors and reviewers again for their time and effort that have put into improving our manuscript. We have addressed all the comments as detailed on our point-by-point response below. For clarity, we first list the reviewers’ comments, our responses are underneath each comment highlighted in yellow background.

**Reviewer #1**:

To ensure reproducibility and to facilitate accurate interpretation by readers, the Figure legends for Figures 4 and 7 should explicitly state the sample size (n) for each group, the statistical methods used, the definition of statistical significance or the exact p values, as well as the units of each mechanical parameter and a brief clarification of what each parameter represents. In addition, a typographical error in the term stiffness is present in Figure 7 and should be corrected.
These issues represent relatively minor revisions and do not affect the conclusions of the study; however, addressing them would substantially improve the completeness and interpretability of the figures. Furthermore, for the other figures in the manuscript, the statistical analysis methods and the use (or absence) of post hoc tests should be clearly stated either in the Methods section or in the corresponding Figure legends.

We would like to thank the reviewer for these suggestions. We fixed the error in Figure 7, and add additional information in Statics and legends of Figures 4 & 7.

Reviewer #2: Revised manuscript has significantly been improved. However, one critical point remains unsolved. In the comment 2) in the previous review, I asked the authors to analyze all the cell lineage for possible changes in cilia (or at least IFT88 expression) in the animals used. The authors seem to have misunderstood the question. Because osterix expression is not "osteoblast-specific," the authors should examine primary cilia in bone cells, at least in cells of the chondrocyte lineage known to express osterix. Osteocytes can also be affected. If IFT88 gene has also been deleted in chondrocytic lineage cells, osteoblasts may not be totally responsible for the observed phenotype. Accordingly, the frequently used phrase "osteoblast-specific" should be softened in the text, and the leaky nature of osterix promoter should be explicitly described as a limitation.

We agree that Osterix expression is not strictly osteoblast-specific and that Cre activity has been reported in chondrocyte lineage cells and, to a lesser extent, osteocytes. We apologize for misunderstanding the reviewer’s original question and appreciate the opportunity to clarify this point.

Importantly, our interpretation is supported by prior work demonstrating that IFT88 deletion in Prx1- or Osterix-expressing cells impairs bone formation and mechanosensitive responses, consistent with a critical role for primary cilia in osteoblast-lineage cells. Nevertheless, we agree that the observed phenotype may reflect combined effects of ciliary dysfunction in multiple Osterix-expressing lineages rather than osteoblasts alone.

In response to the reviewer’s comment, we have revised the manuscript to discuss the potential contribution of chondrocytes and osteocytes to the phenotype in the Discussion section at Page 21. We believe these revisions provide a more accurate and appropriately cautious interpretation of our findings.
